# Advantages and Challenges of Cardiovascular and Lymphatic Studies in Zebrafish Research

**DOI:** 10.3389/fcell.2019.00089

**Published:** 2019-05-28

**Authors:** Massimo M. Santoro, Monica Beltrame, Daniela Panáková, Arndt F. Siekmann, Natascia Tiso, Marina Venero Galanternik, Hyun Min Jung, Brant M. Weinstein

**Affiliations:** ^1^Laboratory of Angiogenesis and Redox Metabolism, Department of Biology, University of Padua, Padua, Italy; ^2^Dipartimento di Bioscienze, Università degli Studi di Milano, Milan, Italy; ^3^Electrochemical Signaling in Development and Disease, Max Delbrück Center for Molecular Medicine, Helmholtz Association of German Research Centers (HZ), Berlin, Germany; ^4^German Centre for Cardiovascular Research: DZHK, Berlin, Germany; ^5^Max Planck Institute for Molecular Biomedicine, Münster, Germany; ^6^Cells in Motion Cluster of Excellence (CiM), University of Münster, Münster, Germany; ^7^Department of Cell and Developmental Biology and Cardiovascular Institute, Perelman School of Medicine at the University of Pennsylvania, Philadelphia, PA, United States; ^8^Laboratory of Developmental Genetics, Department of Biology, University of Padua, Padua, Italy; ^9^Division of Developmental Biology, Eunice Kennedy Shriver National Institute of Child Health and Human Development, Bethesda, MD, United States

**Keywords:** zebrafish models, vascular, lymphatic, heart, trangenesis

## Abstract

Since its introduction, the zebrafish has provided an important reference system to model and study cardiovascular development as well as lymphangiogenesis in vertebrates. A scientific workshop, held at the 2018 European Zebrafish Principal Investigators Meeting in Trento (Italy) and chaired by Massimo Santoro, focused on the most recent methods and studies on cardiac, vascular and lymphatic development. Daniela Panáková and Natascia Tiso described new molecular mechanisms and signaling pathways involved in cardiac differentiation and disease. Arndt Siekmann and Wiebke Herzog discussed novel roles for Wnt and VEGF signaling in brain angiogenesis. In addition, Brant Weinstein’s lab presented data concerning the discovery of endothelium-derived macrophage-like perivascular cells in the zebrafish brain, while Monica Beltrame’s studies refined the role of Sox transcription factors in vascular and lymphatic development. In this article, we will summarize the details of these recent discoveries in support of the overall value of the zebrafish model system not only to study normal development, but also associated disease states.

## Introduction

Zebrafish has been utilized over the past years to successfully study cellular and genetic processes regulating the development of cardiac, vascular, and lymphatic development (Liu and Stainier, 2012; Hogan and Schulte-Merker, 2017; Mauri et al., 2018). Among the several advantages of the zebrafish model system is the large number of embryos that each mating generates (an extraordinary resource for further experimental needs), external development (e.g., easy accessibility for experimental manipulation and imaging), and optical clarity. Distinctively, the optical clarity of zebrafish embryos allows to easily perform live imaging of cells and to track them in the context of a whole organism. An advantage of this feature is the use of transgenic zebrafish animals marking cardiac, blood, endothelial, and lymphatic endothelial cells with genetically encoded markers. Within the last decade, a considerable number of zebrafish fluorescent transgenic lines have been generated to better characterize different components of the vascular system, such as myocardial and endocardial cells, arteries, veins, and lymphatic tissues. The availability of tissue-specific transgenic lines and advanced microscopy techniques (i.e., light sheet and confocal microscopy), have permitted high-resolution time-lapse imaging of developing heart structures, lymphatic and blood vessels, as well as perivascular cells at the subcellular level (Isogai et al., 2001; Huisken and Stainier, 2009; Betz et al., 2016). Such dynamic imaging allows for the dissection of the mechanisms underlying heart and blood/lymphatic vessel patterning, branching, and their regulation.

Furthermore, zebrafish are amenable to both forward genetic approaches (e.g., ENU mutagenesis screens), and reverse genetic studies (such as TALEN and CRISPR/Cas9) (El-Brolosy and Stainier, 2017; Housden et al., 2017). Cre/lox recombinase-based genetics to delete genes of interest in a tissue-specific manner has also been successfully applied to study cardiovascular development and homeostasis (Carney and Mosimann, 2018). Here, we will review the most recent methods and studies on cardiac, vascular, and lymphatic development presented at the recent 2018 European Zebrafish Principal Investigators Meeting in Trento, Italy.

## Recent Advancements on Cardiac, Vascular, and Lymphatic Research in Zebrafish

### Heart Studies

The heart is the first functional organ to form in the vertebrate embryo. While already pumping and maintaining constant blood flow, the heart undergoes profound morphogenetic changes to acquire its final shape. The periodic changes in the mechanical forces as well as intracellular calcium concentrations contribute considerably to the final organ form and function. From the specification of cardiac progenitors, the genetic programs controlling heart formation and function, to cell biological processes of heart morphogenesis, researchers in the zebrafish field have contributed significantly to our understanding of heart development over the last decade (Bakkers, 2011; Staudt and Stainier, 2012). Although several mutants affecting cardiac function have already been isolated during the first large-scale mutagenesis screens (Stainier et al., 1996), the functional patterning of the developing myocardium is a less explored area of research. This is partially due to the lack of *in vivo* techniques to study action potential propagation or changes in intracellular calcium concentrations during each heartbeat. High-speed optical mapping techniques are currently limited to an *ex vivo* analysis (Panáková et al., 2010), however, the growing number of fast genetically encoded voltage and calcium indicators, together with the recent advances *in vivo* imaging (Weber et al., 2017), will allow researchers to close this gap of knowledge in the very near future.

Pertaining to the functional patterning of the developing myocardium, Dr. D. Panáková expanded on already reported data showing the Wnt11 non-canonical pathway regulating the intercellular electrical coupling in the developing zebrafish heart through attenuation of the L-type calcium channel (LTCC) conductance (Panáková et al., 2010). The LTCC has an important function in cardiac biology. Its conductance is regulated by membrane depolarization or by the β-adrenergic/Protein kinase A (PKA) pathway, and mediates Ca^2+^ influx. The molecular mechanisms by which Wnt11 signaling might modify LTCC, and thus regulate its activity, were unclear. The research group of Dr. Panáková found that Wnt11 signaling regulates the LTCC through its effects on the A-kinase anchoring protein (AKAP)/PKA signalosome. They further showed that the AKAP/PKA signalosome is an essential component of Wnt11 signaling regulating channel function during the establishment of intercellular electrical coupling gradients in the developing zebrafish heart.

The field of arrhythmias is indeed another exciting area where zebrafish models have been successfully applied, as demonstrated by the pioneering work of the MacRae and Saffitz groups (Milan and MacRae, 2008; MacRae, 2010; Asimaki et al., 2014). In the specific context of inherited arrhythmogenic cardiomyopathies (AC), mostly linked to defects in desmosomal components (Pilichou et al., 2016), Dr. Tiso’s group presented the first zebrafish model for human AC type 8 (AC8), a form of AC caused by mutations in the desmosomal protein desmoplakin (Rampazzo et al., 2002). By knocking down both zebrafish desmoplakin genes (*dspa* and *dspb*) Tiso’s team could specifically phenocopy the ultrastructural features and cardiac frequency defects of AC8. Moreover, the availability of zebrafish lines, which are able to report *in vivo* the activation of a series of signaling pathways (Moro et al., 2013), was exploited in the study to analyze a set of signals known to be involved in cardiac function.

The screen allowed to identify three pathways (Wnt/beta-catenin, Hippo/YAP-TAZ, and TGFbeta), out of nine considered, that are specifically altered in AC8-like conditions (Giuliodori et al., 2018). Interestingly, in chronic organ injury, a possible cross-talk among these signals has been recently hypothesized (Piersma et al., 2015). This discovery, made in a zebrafish setup, is thus offering a new, network-wide view of AC conditions, and suggesting a set of evolutionary conserved signaling mechanisms worth to be considered in the design of a molecularly-targeted therapy for AC.

### Vascular Studies

As a model system, the zebrafish offers unique advantages for studying vascular development *in vivo* (Hogan and Schulte-Merker, 2017). Recent findings have also shown that the zebrafish possesses a *bona fide* brain vascular system, making it useful for studying the formation of this distinct vascular network in the head. In Trento, Dr. Wiebke Herzog and Dr. Arndt Siekmann provided new insights on the mechanisms that govern the formation of the brain vasculature in early zebrafish embryos. It is well established that Vascular Endothelial Growth Factor A (VEGFA) is one of the main signaling molecules important for blood vessel growth and homeostasis (Simons et al., 2016). Work over the last years has provided detailed insights on the downstream signaling events that occur upon receptor activation (Alvarez-Aznar et al., 2017). However, it has remained enigmatic how endothelial cells balance these diverse signaling outcomes in order to temporally and spatially control initiation of cell migration and proliferation. Instead of a single *VEGFA* gene, zebrafish possess two VEGFA genes, *vegfaa* and *vegfab* (Bahary et al., 2007). Dr. Arndt Siekmann presented data on the different phenotypes of *vegfaa* and *vegfab* mutants. As expected, *vegfaa* mutants displayed severe brain blood vessel defects. Surprisingly, *vegfab* mutations mainly affected endothelial cell proliferation. Blood vessels could still sprout and form a relatively normal vascular architecture, despite a reduction in endothelial cell numbers. These findings indicate that proliferation and migration can be uncoupled during angiogenic blood vessel sprouting. They furthermore suggest an unexpected plasticity in terms of endothelial cell sizes when it comes to establishing vascular networks. The important next steps will be to identify *vegfab* targets mediating cell cycle progression, but not cell migration.

Another signaling pathway regulating brain blood vessel formation is Wnt signaling (Reis and Liebner, 2013). Mutations in the Wnt pathway components Wnt7 (Stenman et al., 2008), GPR124, and Reck (Vanhollebeke et al., 2015; Ulrich et al., 2016) were previously shown to specifically interfere with brain blood vessel formation. Of note, blood vessel formation in other regions of the embryos can proceed relatively unperturbed in these mutants. This suggests that the brain vasculature requires a distinct set of molecules for its formation. One reason for this might be the necessity to also form the blood brain barrier during embryonic development that shields the brain. New results from the laboratory of Dr. Herzog now provide evidence that Wnt signaling is less required for the initial sprouting of brain blood vessels, but is rather necessary in newly connecting blood vessels. Here, Wnt signaling regulates the localization of junctional proteins in order to facilitate blood vessel anastomosis. Thus, Wnt signaling might coordinate the different steps necessary for the proper development and maturation of brain blood vessels. Further experiments will aim at a better understanding of the molecules that mediate these different effects of Wnt signaling.

### Lymphatic Studies

Mammals, zebrafish, and other vertebrates possess a second vascular system entirely separate from the blood circulatory system called the lymphatic system. The lymphatic system is a complex, blind-ended vascular network that extends into virtually every part of the body. It is essential for maintaining fluid homeostasis, absorbing dietary lipids from the intestine, and for immune cell production and trafficking (Baluk et al., 2007; Alitalo et al., 2005). Since the first identification of zebrafish lymphatic vessels in 2006 (Kuchler et al., 2006; Yaniv et al., 2006), a number of studies have provided evidence that the zebrafish lymphatic system shares many of the morphological, molecular, and functional characteristics of lymphatic vessels in other vertebrates (Yaniv et al., 2006; Hogan et al., 2009; Nicenboim et al., 2015; Jung et al., 2017). The zebrafish has helped identify important molecular players in lymphangiogenesis such as *cxcr4-cxcl12* chemokine signaling, which provides guidance cues directing early trunk lymphatic development (Cha et al., 2012), and *pkd1* for polarity, elongation, and the adherens junctions for lymphatic endothelial cells that are essential for vessel morphogenesis (Coxam et al., 2014). The Weinstein lab recently generated a valuable new *Tg*(*mrc1a:egfp*)*^y251^* transgenic zebrafish line for visualizing and investigating lymphatic vascular development (Jung et al., 2017). The Mannose receptor (MR, CD206 or MRC1) is a transmembrane glycoprotein that belongs to the C-type lectin family, known to be strongly expressed in macrophages, dendritic cells, and prominently in lymphatics (Taylor et al., 2005). MRC1 is involved in lymphocyte migration (Marttila-Ichihara et al., 2008). Zebrafish *mrc1a* is expressed in primitive veins and lymphatics (Wong et al., 2009; Jung et al., 2017), and this new transgenic line has proven extremely useful for studying the growth and assembly of novel major superficial and deep lymphatic vessels in the trunk that form during later stages of development (Jung et al., 2017).

In the course of using the *Tg*(*mrc1a:egfp*)*^y251^* transgenic line, the Weinstein lab researchers discovered that in addition to lymphatics, this transgenic line also displays strong EGFP expression in a novel cell population on the surface of the zebrafish brain (Venero Galanternik et al., 2017). Despite expressing *mrc1a:egfp* and other lymphatic transgenes, these newly discovered brain cells are individual non-lumenized perivascular cells, associated with blood vessels located exclusively in the brain meninges. In addition to a macrophage-like morphology, these cells also contain large numbers of internal auto fluorescent vesicles or vacuoles, and they exhibit extremely robust scavenger activity as shown by rapid uptake of India Ink and other “tracers” injected into intracranial spaces or brain ventricles. Careful cell lineage and live imaging studies demonstrated that these cells emerge by transdifferentiation from the venous endothelium of the optic choroidal vascular plexus, and RNA-seq on FAC-sorted Mrc1a-positive cells revealed that despite their macrophage-like morphology and perivascular location, they appear molecularly most similar to lymphatic endothelium, strongly expressing lymphatic markers such as *lyve1, prox1a*, and *flt4* (Venero Galanternik et al., 2017). The expression of a number of different lymphatic transgenes also led two other groups to contemporaneously discover and report on these cells in the zebrafish (Bower et al., 2017; van Lessen et al., 2017). Based on similarities in their morphology, location, and scavenger behavior, these cells appear to be the zebrafish equivalent of cells variably characterized as Fluorescent Granular Perithelial cells (FGPs), perivascular macrophages, or “Mato Cells” in mammals (Mato et al., 1984). In mammals, FGPs are found in the leptomeningeal layers surrounding blood vessels, where they are thought to provide an important pinocytotic protective function. Further study of FGPs in the experimentally and genetically accessible zebrafish model system is sure to yield important new insights into blood-brain barrier establishment, removal of toxic waste from the brain, and the relationship between brain homeostasis and waste clearance and neurodegenerative disorders and protection from brain injury and infection.

A debate has been going on in recent years, within the zebrafish community, on the use of morpholinos to study gene function, based on the published observation that mutants in several genes, obtained through reverse genetic approaches, were not sharing the same phenotypes of the corresponding morphants (Kok et al., 2015). Genetic compensation elicited in mutants, but not in morphants, rather than simply toxicity or off-target effects of morpholinos, might be at the basis of these discrepancies (Rossi et al., 2015), and not only in zebrafish, but also in other model organisms where knockout and knockdown approaches are giving different results (El-Brolosy and Stainier, 2017). Cermenati et al. (2013) showed, through the use of morpholinos, that the transcription factor Sox18 plays a role in early zebrafish lymphatic development, suggesting an evolutionary conservation between fish and mammals. Mutations in *SOX18* underlie both recessive and dominant forms of Hypotrichosis-Lymphedema-Telangiectasia syndrome (Irrthum et al., 2003); the phenotypic spectrum is highly variable among patients and most, but not all of them, present with lymphedema (Moalem et al., 2015; Wunnemann et al., 2016). *Sox18*-null mice develop severe lymphedema in a pure B6 background, but show no signs of lymphatic dysfunction in a mixed background, where the upregulation of the closely related transcription factors Sox7 and Sox17 in the cardinal vein compensates for the lack of Sox18 (Francois et al., 2008; Hosking et al., 2009).

van Impel et al. (2014) reported that in a *sox18* loss-of-function mutant thoracic duct (TD) formation was unaffected and they questioned the role of Sox18 in the regulation of lymphatic development in zebrafish. Dr. Monica Beltrame presented at the meeting data gathered in her lab on an independent *sox18*-null mutant, generated through the Zebrafish Mutation Project at Sanger (Kettleborough et al., 2013). Homozygous *sox18* mutants showed subtle but statistically significant defects in TD formation (Moleri et al., unpublished data). A slight perturbation of VegfC signaling exacerbated TD defects in a genotype-dependent manner, unraveling differences in TD formation even in *sox18* heterozygotes versus wild types. Remarkably, an upregulated expression of *sox7* in the posterior cardinal vein was observed in *sox18* mutants, thus possibly explaining the milder lymphatic phenotypes with respect to the knockdown of *sox18*, which was not affecting *sox7* expression (Cermenati et al., 2013). Overall, a high degree of evolutionary conservation in the regulation of lymphatic development by SoxF TFs is thus confirmed.

## Reporter Tools and Genetic Versatility for Cardiovascular Studies in the Zebrafish System

In recent years, cardiovascular research in zebrafish greatly benefited from the development of a series of innovative genetic tools, mostly represented by mutant, transgenic, and biosensor lines, allowing to analyze *in vivo* a wide range of components in physiological as well as in pathological contexts (development, signaling, contraction, regeneration, inflammation) occurring in the cardiovascular compartment. To date, several zebrafish lines have been generated to identify and perturb *in vivo* different sub-cardiac compartments, such as the endocardium, myocardium, epicardium, valves, or conduction system as well as specific vascular components and cell types including arteries, veins, lymphatic vessels, mural, and endothelial cells (Santoro et al., 2009; Bakkers, 2011; Gore et al., 2012; Bournele and Beis, 2016).

In this perspective, during the EZPM2018 in Trento, a spectacular series of new interesting systems have been presented, all worth to be reported and added to the available toolkit. In the context of new mutant and transgenic line production, the research group of Dr. Beltrame presented a new allele for Sox18, which allowed to further elucidate the role of this key factor in the development of lymphatic vessels in vertebrates. Dr. Siekmann’s research group has produced new mutant lines for the two *VEGFA* paralogs (*vegfaa* and *vegfab*), elegantly demonstrating that both factors act through the same receptor (Kdrl) to induce sprouting, vessel formation, and vascular-specific gene expression. Finally, using the transgenic line *Tg*(*mrc1a:eGFP*), Dr. Weinstein’s group could efficiently analyze a new population of perivascular cells *in vivo* (Jung et al., 2017).

In the field of cardiovascular signaling, Dr. Tiso’s group presented the systematic application of pathway-specific transgenic lines for the identification of drugs with therapeutic effects in cardiac pathology. Specifically, a form of arrhythmogenic cardiomyopathy was modeled in a set of different zebrafish lines reporting signaling pathways activated in the cardiac compartment. This signaling-based screen allowed identifying the involved cascade, rapidly passing to the test of pathway-specific compounds, and the identification of a Wnt agonist as a potential therapeutic drug (Giuliodori et al., 2018). The role of the canonical Wnt pathway in the vascular compartment has also been studied by Dr. Herzog’s group (Hubner et al., 2017). In particular, her team produced and presented a set of transgenic lines able to express in a vascular-specific way a dominant negative form of a Wnt effector (dnTcf), as well as destabilized Wnt-responsive fluorophores. With the combined use of these systems, the team could successfully inhibit Wnt signaling in a temporal and tissue-specific fashion, analyzing the response to the pathway within time frames of about 2 h. This strategy allowed to establish a new role for the Wnt pathway in the anastomosis of the capillaries during the formation of the blood brain barrier.

## Challenges and Outlook

Zebrafish is a long-standing model to study heart morphogenesis. With the growing number of genetic tools and technological advances, probing the functional inputs and their related physical stimuli, such as mechanical or calcium cues in greater detail *in vivo*, will soon ensue. The dysregulation of ion channels, such as LTCC, can result in arrhythmias as well as cardiac hypertrophy, both of which are underlying pathologies leading to sudden cardiac death. Future studies, in which modulation of functional inputs plays a crucial role, will therefore also accelerate novel avenues for future therapies regarding common cardiac diseases, including cardiac hypertrophy, heart failure, and arrhythmias.

Many questions still remain open in the field of vascular and lymphatic studies in zebrafish. Besides VEGF and Wnt, other signaling pathways are increasingly studied in vascular development, such as the Hippo pathway (Nagasawa-Masuda and Terai, 2017; Nakajima et al., 2017; Astone et al., 2018). Similarly, hemodynamic forces seem to play a critical role in cardiovascular development and differentiation (Chen et al., 2012, 2017; Goetz et al., 2014).

We envision a new interactive atlas of zebrafish vascular and lymphatic anatomy using modern technologies, such as light sheet microscopy and fluorescent markers of different colors. It would be a *desideratum* for researchers working in the field to also include perivascular cells, such as mural cells.

It can be expected that in the coming years the growing application of single-cell sequencing technologies and the use of reporter pathway lines, with conditional modulation, combined with mutant lines for new regulators of cardiac as well as vascular components, will allow a highly specific genetic dissection of each process, with thus far unprecedented precision in the cardiovascular research field. We expect that cutting-edge high throughput approaches, such as single-cell RNA sequencing and metabolomics will be feasible in the near future (Santoro, 2014; Pandey et al., 2018). We envision that the zebrafish model system will continue to help in understanding the genetic and molecular basis of human cardiovascular and lymphatic malformations and diseases.

## Data Availability

The datasets generated for this study are available on request to the corresponding author.

## Author Contributions

All authors contributed to the writing of the manuscript. MMS coordinated the work.

## Conflict of Interest Statement

The authors declare that the research was conducted in the absence of any commercial or financial relationships that could be construed as a potential conflict of interest. The handling Editor declared a past co-authorship with one of the authors NT.
